# Supplementary material for: Meroterpenoids with Immunosuppressive Activity from Edible Fungus Craterellus odoratus
Source: Molecules. 2023 Feb 6;28(4):1564. doi: 10.3390/molecules28041564 (PMC9963684; doi:10.3390/molecules28041564)
Supplement: Supplementary file 1 [file molecules-2176637-supplementary (4) (1).pdf › molecules-2176637-supplementary (4) (1).pdf]

Supporting Information for

Meroterpenoids with immunosuppressive activity from edible fungus *Craterellus odoratus*

Quan Dai [1,a], Li-Ting Pang, [1,a] Fa-Lei Zhang [1], Gang-Qiang Wang [2], Xue-Mei Li [3], Ji-Kai Liu [1,*] and Tao Feng [1,*]

[1] School of Pharmaceutical Sciences, South-Central Minzu University, Wuhan 430074, China
[2] Hubei Key Laboratory of Radiation Chemistry and Functional Materials, Non-power Nuclear Technology Collaborative Innovation Center, School of Nuclear Technology and Chemistry & Biology, Hubei University of Science and Technology, Xianning 437100, China
[3] Plant Protection and Quarantine Station of Dehong, Dehong 678400, China
[a] These authors contribute equally to this work.
[*] Correspondence: tfeng@mail.scuec.edu.cn (T. F.); liujikai@mail.scuec.edu.cn (J. K. L)

Contents

Section S1. Calculational details for **1**
Section S2. Calculational details for **2**
Section S3. NMR and MS spectra of **1**
Section S4. NMR and MS spectra of **2**
